# Social Diversification Driven by Mobile Genetic Elements

**DOI:** 10.3390/genes14030648

**Published:** 2023-03-04

**Authors:** Michael L. Weltzer, Daniel Wall

**Affiliations:** Department of Molecular Biology, University of Wyoming, Laramie, WY 82071, USA

**Keywords:** kin discrimination, evolution, horizontal gene transfer, mobile genetic elements, social diversification

## Abstract

Social diversification in microbes is an evolutionary process where lineages bifurcate into distinct populations that cooperate with themselves but not with other groups. In bacteria, this is frequently driven by horizontal transfer of mobile genetic elements (MGEs). Here, the resulting acquisition of new genes changes the recipient’s social traits and consequently how they interact with kin. These changes include discriminating behaviors mediated by newly acquired effectors. Since the producing cell is protected by cognate immunity factors, these selfish elements benefit from selective discrimination against recent ancestors, thus facilitating their proliferation and benefiting the host. Whether social diversification benefits the population at large is less obvious. The widespread use of next-generation sequencing has recently provided new insights into population dynamics in natural habitats and the roles MGEs play. MGEs belong to accessory genomes, which often constitute the majority of the pangenome of a taxon, and contain most of the kin-discriminating loci that fuel rapid social diversification. We further discuss mechanisms of diversification and its consequences to populations and conclude with a case study involving myxobacteria.

## 1. Introduction

Evolution is a story of diversification. It essentially involves changes in lineage genomes that may eventually result in new taxa. Different selective forces drive diversification and impactful changes, made by mutations, recombination or horizontal gene transfer (HGT), are maintained provided there is a fitness benefit [1]. Environmental factors, such as spatial separation, distinct ecological niches, competition and other forces, including genetic drift, promote diversification. Central to this is the formation of new social groups, where individuals within a group cooperate, but not with those that belong to other groups. Social diversification in turn can lead to recombination barriers that ultimately facilitate the evolution of distinct taxa. 

Social groups consist of cooperative interactions between related individuals that lead to fitness gains, which are often not attainable by individuals [2,3]. However, when cooperation is blocked, and/or antagonism is initiated, diversification ensues. In microbes, social diversification is directly linked to gene content changes. In bacteria, this is frequently caused by HGT. This review focuses on the role of HGT in social diversification and, in particular, how mobile genetic elements (MGEs) serve as powerful engines of diversification. Here, the acquisition of new genes, or their loss, results in cells that no longer productively cooperative with their recent siblings. Instead, such diversification typically leads to antagonism between related individuals, herein broadly defined as kin discrimination (KD). In turn, social conflict itself can lead to accelerated evolution manifested as arms races or Red Queen evolution [4]. These arms races likely explain why many bacterial genomes contain a large number of KD genes [5,6,7,8,9,10,11]. Tied to diversification, we also discuss the dynamic nature of bacterial genomes and mechanisms that govern KD.

### 1.1. Intraspecific Genome Diversity and Fluidity

The dynamic nature of prokaryotic genomes typically results in an exceptionally high degree of intraspecific diversity. This diversity is readily apparent when comparing genomes within a species and assessing pangenome content, which represents all genes found within a taxon [12]. In most cases, pangenomes mostly consist of accessory genes that are present in only a subset of lineages. For some species, their core genomes, genes found in all isolates, only represent <20% of their pangenome [12,13,14]. Many of these accessory genes are derived from MGEs, which themselves can represent >25% of a genome [15]. As highlighted here, MGEs play key roles in social diversification, in part because they are the most dynamic class of genes in prokaryotes [16]. 

Although bacterial genomes are fluid, where new genes are added by HGT, the sizes of genomes within a species typically remain relatively constant [17]. This is partly explained by gene addition being countered by gene loss. More broadly, in the absence of HGT and selective pressures, the prevailing mode of change is by genome contraction [18], where the compact nature of bacterial genomes is caused by a mutational bias toward deletions [14,19]. In fact, the rate of deletion to insertion is favored about 10:1 [20], and thus DNA that does not benefit the host is lost over time, as dramatically revealed by the small genomes of many obligate intracellular parasites [19]. Additionally, microbes can adapt to environmental changes by gene loss, leading to the ‘less-is-more hypothesis’ [21,22]. In support of this, a meta-analysis of studies on *E. coli* MG1655 grown in 144 different environmental conditions determined that adaptive null mutations had occurred in 139 of the 144 conditions [23]. Strikingly, under nutrient deprivation, adaptive null mutations were found that nearly doubled the growth rate [22,23].

**Figure 1:**
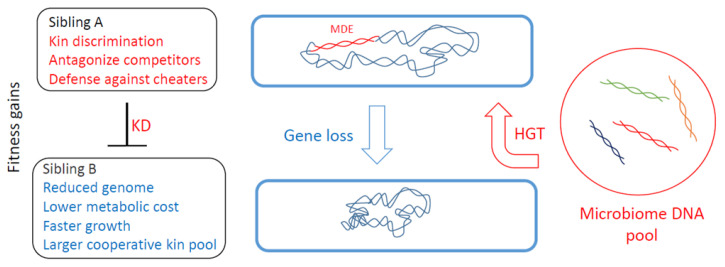
Genome fluidity and social evolution. Top cell receives mobile discrimination element (MDE) from the environment, resulting in sibling A antagonizing sibling B. However, after many rounds of cell division, a daughter cell may lose the MDE, rendering it susceptible to its ancestral population, but nevertheless contributing toward a reduced genome and resulting in different fitness gains.

**Figure 2:**
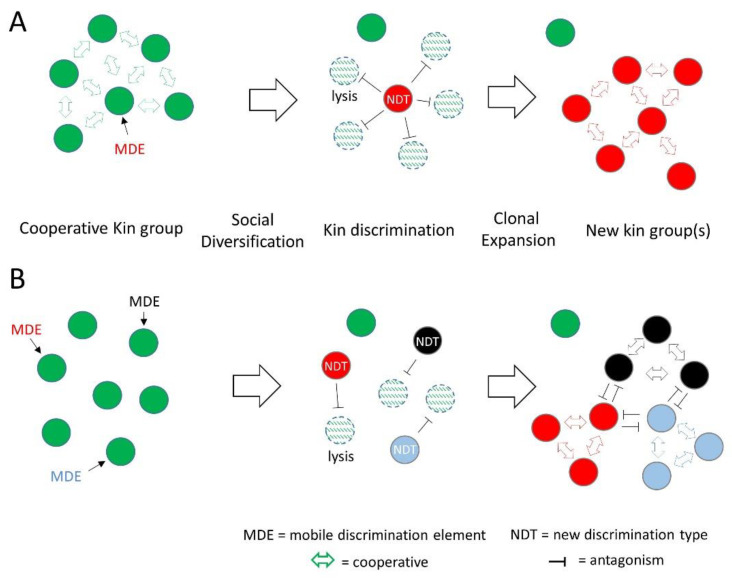
Social diversification mediated by HGT of MDEs. (**A**) A cell in a clonal population is transformed with an MDE containing a novel toxin-immunity locus that creates a new discrimination type (NDT; different color), which antagonizes siblings and overtakes the population. (**B**) Multiple cells in a clonal population transformed by different MDEs. These NDTs antagonize their siblings, resulting in new social groups that likely antagonize each other and do not cooperate.

**Figure 3:**
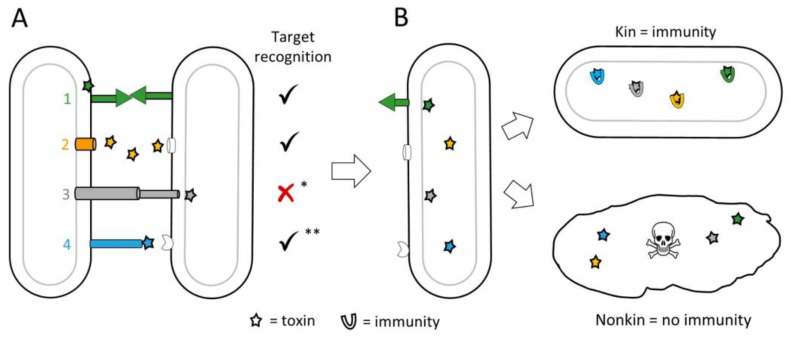
Mechanisms of kin discrimination. (**A**) Toxin delivery by four general strategies: 1. Homotypic binding between compatible receptors. In myxobacteria, this occurs between TraAB receptors triggering OME and delivery of a suite of SitA lipoprotein toxins. 2. Cell releases diffusible bacteriocins/tailocins that target distant relatives. 3. Cell-contact-dependent secretion systems, e.g., T6SS, deliver toxins to target cell. 4. Toxin displayed on distal end of a protruding cell surface protein, e.g., CDI, targeting a receptor on kin. * Recognition not required but can occur; ** required for CDI, unknown for very large class of Rhs proteins found in myxobacteria. Check mark means yes; red *x* means no. (**B**) Delivered toxins either neutralized by cognate immunity factors in close kin, e.g., clonemates, or poison distant relatives lacking immunity.

Although MGEs are selfish entities that replicate for their own gain, they also carry traits that impact the host. Many of these traits influence how the host interacts with others, including means for KD, which we describe as mobile discriminating elements (MDEs). Genes that govern interactions with kin are by definition social genes, and the constellation of social genes determines the degree of relatedness between individuals [3], as expressed by Hamilton’s rule (see below) [24]. Thus, the acquisition or loss of MDEs changes an individual’s social relationships and overall fitness (Figure 1). Moreover, the acquisition of a new MDE can confer an adaptive benefit whereby the individual discriminates against siblings and thus creates a new social group (Figure 2). In these scenarios, the new discriminatory behavior might be driven by a single locus, e.g., a toxin-immunity cassette. In such cases, this behavior can be called kind or greenbeard discrimination to highlight its genetic simplicity [2]. However, in many cases, other loci also contribute towards KD, such as genes required for toxin delivery (Figure 3).

### 1.2. Benefits of Kin Discrimination

Determining genetic relatedness between individuals allows microbes to form sustainable social interactions. That is, individuals need to discern close genetic relatives or siblings that cooperate with themselves from distant microbes with similar metabolic needs that are competitors or social cheaters. Although microorganisms are often thought of as autonomous unicellular organisms, cooperative and even altruistic behaviors are abundant in microbial populations. These behaviors range from secreting a shared public good to self-sacrifice, e.g., autolysis. Evolutionary theory and experimental work have addressed how social behaviors evolve, given a seemingly contradictory Darwinian framework that individuals only behave in their own self-interests. Hamilton [24] proposed that cooperative and altruistic behaviors evolve when they are directed toward close kin that share social genes. Hamilton’s rule states that *rb* > *c*, where *r* is the genetic relatedness between the actor and the recipient, *b* is the benefit to the actor, and *c* is the cost to the actor. Thus, an altruistic behavior that has a fitness cost to the actor can evolve if it provides a fitness benefit to a sufficient number of close relatives that share social alleles. This rule was summarized by evolutionary biologist JBS Haldane’s comment that he would not lay down his life for a brother, but would for two brothers or eight cousins [25]. In support of Hamilton’s rule, a meta-analysis of twelve studies found that altruism is often under positive selection [26]. Since altruistic behavior must be directed towards close kin to evolve, organisms need mechanisms to determine their genetic relatedness to other individuals. 

In many cooperative systems, social cheating arises. A cheater is an individual that reaps the benefit of a cooperative action but incurs reduced or no cost to itself. If cheaters expand in a population, it leads to the loss of the cooperative trait and collapse of the social system [27,28,29]. Similarly, within multicellular organisms, such as animals, cancer cells cheat by exploiting their cooperative cellular environments, which often leads to the demise of the individual. Examples of cheaters in biofilms are individuals that that use public secreted goods produced by others, but they do not contribute themselves. Cheaters also arise in aggregative multicellular organisms, such as myxobacteria and social slime molds. In myxobacteria, >60% of cells lyse during fruiting-body development, while less than 20% form viable spores [30]. The lysed cells apparently represent an altruistic behavior that provides nutrients to the starving population and consequently supports the metabolically demanding process of spore formation, which occurs over days [31]. However, cheaters exploit this cooperative environment and become disproportionately represented in spores and thus do not undergo their fair share of lysis [31,32,33,34,35,36]. Similarly, in *Dictyostelium discoideum* fruiting bodies, cheaters arise in which they are over-represented among spores, and under-represented in terminally differentiated stock cells [37,38,39]. Because there are inherit selective advantages for cheater cells to arise, species need mechanisms to protect their cooperative traits from exploitation. To do so, organisms use self-recognition and/or KD systems [40,41].

As mentioned, secreted goods are particularly vulnerable to cheating, because once they leave the producer, any neighboring cell can exploit them. An alternative strategy is for cells to share goods privately. An example of shared private goods comes from outer membrane exchange (OME) in myxobacteria [42]. In this system, individuals recognize one another through homotypic binding between compatible (e.g., identical) TraA receptors, which are highly polymorphic and hence provide specificity for kin recognition [43,44]. After receptor binding, cells exchange portions of their outer membranes and proteins by an apparent transient membrane fusion event(s) [45]. This behavior is hypothesized to evolve as a mechanism to protect cooperative interactions. 

### 1.3. KD Mechanisms Are Plentiful and Diverse

Given the importance of KD, it is not surprising that these systems are widespread and diverse throughout the bacterial kingdom [2,3,7,46,47,48]. Here we give a brief overview of antagonism- and non-antagonism-based KD systems. Although many of the examples given are associated with MGEs, there are also many cases where KD loci are separate from MGEs.

KD systems frequently contain toxin-immunity gene cassettes where an individual delivers effectors to neighboring cells, which only have immunity if they are related or clonal cells (Figure 3). In the case of myxobacteria, which display extensive multicellular and cooperative interactions [49], they use multi-layered KD systems. As described, the first level of kin recognition is mediated by binding of compatible TraA receptors that trigger OME. During OME, subsets of the exchanged cargo are lipoprotein effectors known as SitA toxins. These toxins function in KD, because only related individuals with compatible TraA receptors receive them [50]. After delivery to the outer membrane, SitA toxins employ their ‘escort domain’ to hijack inner membrane proteins to enter the cytoplasm where they act as nucleases [51]. *sitAI* toxin-immunity gene cassettes are widespread, where some myxobacteria contain >80 loci. In turn, this allows each strain to have its own exquisite ‘self-identity barcode’ consisting of a suite of unique toxin-immunity loci [52]. The exchange of SitA toxins thus serves as a second layer or a verification step for KD [53].

Since OME only functions in KD between strains with compatible TraA receptors [44,50,54], other systems are required. Indeed, when testing environmental *Myxococcus xanthus* isolates [5], the elimination of OME by inactivating *traA* did not prevent antagonism. Instead, to eliminate antagonism, double mutants that knocked out OME and the type VI secretion systems (T6SS) were required, and for two strains, a third system (Rhs) had to be knocked out. This revealed that *M. xanthus* uses parallel KD systems to deliver discriminating effectors. As found with *sitAI*, comparative genomic analysis found divergent T6SS and Rhs effector loci in an MGE called Mx-α, a polymorphic lineage of prophages [50,54,55]. Additionally, Gong et al. [56,57] identified T6SS effectors involved in colony merger incompatibility (KD) between isogenic *M. xanthus* strains. Thus, in one context, T6SS in *M. xanthus* allows KD against myxobacteria of variable degrees of relatedness, while OME allows KD against closely related individuals that share compatible TraA receptors. 

Interestingly, the T6SS also discriminates against siblings that are physiologically less fit [58]. For example, in *M. xanthus*, when a histidine auxotroph is mixed with its isogenic parent, the parent kills the auxotroph in the absence of histidine, but not in the presence of histidine. Here, a phylogenetically conserved T6SS effector mediates sibling antagonism. Mechanistically, antagonism occurs because the level of the immunity protein decreases during histidine starvation, and thus the indiscriminate injection of this T6SS effector discriminates against less fit siblings. 

Similarly, *Bacillus subtilis* uses a combination of KD mechanisms. Here, Lyons et al. [6] proposed that surface receptors, contact-dependent inhibition (CDI) effectors, secreted compounds and mobile elements all function in KD. Kraigher et al. [59] further investigated KD of environmental isolates at a centimeter scale, and found that when closely related strains were mixed, they swarmed together to colonize a surface. However, when genetically divergent strains were mixed, one strain outcompeted the other and colonized the surface alone. In a separate study, Lyons & Kolter [60] investigated *Bacillus* interactions from a broader relatedness scale, i.e., between *Bacillus* species. They found *Bacillus* cells discerned the degree of relatedness, where strong levels of antagonism occurred between closely related species, and low levels of antagonism occurred between distant isolates. Nevertheless, antagonism occurred against distantly related species that produced the same surfactant molecule, an exploitable common good.

In contrast to cell-contact-dependent discrimination, many organisms deploy diffusible discrimination factors. For example, Vacheron et al. [61] found that *Pseudomonas protegens* produce R-tailocins, which, following lysis of the producing cell, are dispersed and specifically target competing cells by puncturing their cell membranes. Other diffusible KD factors commonly found on MGEs include bacteriocins, colicins and antibiotics [2,3,62,63,64]. 

KD also occurs in the absence of lethal toxin delivery. It has long been recognized that when swarms of divergent strains of *Proteus mirabilis* meet, a demarcation zone appears [65]. Genetic analysis determined that the six-gene *ids* locus is responsible for this inhibition [66]. Subsequent studies determined that two cognate proteins, IdsD and IdsE, must interact for kin recognition. IdsD is delivered by T6SS, and if cells are kin, IdsD interacts with IdsE in the recipient, and the effector is neutralized. If cells are nonkin, the cognate IdsE is absent, allowing unbound IdsD to increase levels of the secondary signaling molecule ppGpp, which results in transcriptional changes and decreased swarming. Thus, nonkin cells drop out of a swarm, ensuring that a swarm is composed only of cooperative kin cells [67,68]. However, in other *P. mirabilis* strains, T6SS serves in antagonism delivering lethal toxins [69]. In another example of non-lethal KD, environmental isolates of *Vibrio cholerae* harboring differing variants of the PilA pilin lacked the ability to aggregate, and thus formed distinct social groups [70].

### 1.4. MGEs Harbor Diverse Types of KD Systems

Many KD loci are encoded on MGEs (Table 1). Perhaps the best-known examples are colicin toxins encoded on *Escherichia coli* plasmids (reviewed in [71]), which are secreted into the milieu and kill *E. coli* cells lacking that MGE. Additionally, toxin-delivery systems are also found on plasmids. For instance, Morgado and Vicente [72] identified 330 plasmids, primarily in the Proteobacteria, that encode T6SS genes. T6SS genes are also encoded on integrative conjugative elements (ICEs) and genomic islands, suggesting HGT [72,73]. Recently, a T6SS operon in *Vibrio fischeri* was found that functions in KD [74] and contains a putative lipoprotein that specifies target cells [75]. 

Lysogenic phage are another type of vehicle that disseminates KD loci and thus serve as catalysts for social diversification. For instance, MuF toxins are associated with phages prevalent in the human gut microbiome [76]. In *E. coli*, the formation of colony demarcations, i.e., KD, was found when one strain contains a prophage and the other does not [81]. Here, a sub-population of cells lyse and release phages, resulting in KD against siblings. In *M. xanthus*, *sitAI* and T6SS effector loci are frequently located on prophages and thus discriminate against related strains that lack immunity [5,54]. Additionally, HGT by prophages was proposed to disseminate KD genes in *B. subtilis* [6]. 

CDI systems are widely distributed in several classes of Proteobacteria where they antagonize close kin. In some cases, CDI genes are located in or near transposable element genes, suggesting horizontal transfer [77,80], and are also stably maintained in plasmids that become fixed in populations [82]. In *Bacteroides* and *Parabacteroides* residing in the human gut, ICE families were found, which contained T6SS operons and, strikingly, were horizontally transferred within a person and between related species [78,79]. Moreover, the horizontal transfer of these elements led to fixation of particular strains in at least eight cases in two human gut ecosystems [79]. These findings illustrate how the transfer of MDEs can rapidly change kin groups in an ecosystem, as shown in Figure 2. 

### 1.5. Do MGEs Promote Cooperation?

An intriguing and debated idea is whether MGEs also promote social cooperation [83]. Some argue that cooperation, i.e., public-good sharing, is promoted by HGT, which can also act as a mechanism to guard against cheating. For example, when a secreted good is encoded on an MGE, a cheater that does not secrete the compound, but benefits and co-exists in the population, becomes a likely target for infection by that MGE, thus preventing a cheater from overtaking a population [83,84]. Furthermore, if KD loci are carried on that MGE, it acts as a policing mechanism to punish non-cooperators [83]. These ideas have sparked debates, which are primarily centered on secreted virulence factors encoded on plasmids [83,85,86,87,88].

Nogueira et al. [85] used modeling to show how horizontal transfer promotes cooperation. In support of their model, they analyzed 20 *E. coli* genomes and their plasmids and found that proteins that are secreted or localized in the outer membrane are more likely to be encoded on mobile elements and transfer hotspots. Likewise, other studies concluded that genes encoding secreted goods are overrepresented on plasmids [86]. In contrast, Dewar et al. [88] analyzed 1,632 genomes from 51 species and did not find support of this idea. Instead, they concluded that transfer of MGEs may promote cooperation in the initial invasion of a population, but not for their maintenance.

If HGT is not sufficient to maintain cooperation, then how do these elements persist? One solution comes from the Black Queen Hypothesis [89]. This hypothesis predicts genes that produce costly beneficial public goods will be lost by most individuals, but are retained in just enough individuals to support the population, resulting in dependency between individuals. Put another way, the Black Queen Hypothesis can be viewed as a ‘division of labor’ [83].

Besides plasmids, other MGE types contribute to cooperation, though less is known [83]. In one case, the spread of beneficial genomic islands (GIs) through an aquaculture community was found [90]. By using pangenome sequencing of *Vibrio parahaemolyticus*, GIs encoding virulence genes and antibiotic resistance genes were discovered. Some of these GIs were also found in other *Vibrio* taxa that interact positively with *V. parahaemolyticus*, suggesting a cooperative function mediated by HGT. 

### 1.6. Does Social Diversification Benefit Bacterial Populations at Large?

**Figure 4:**
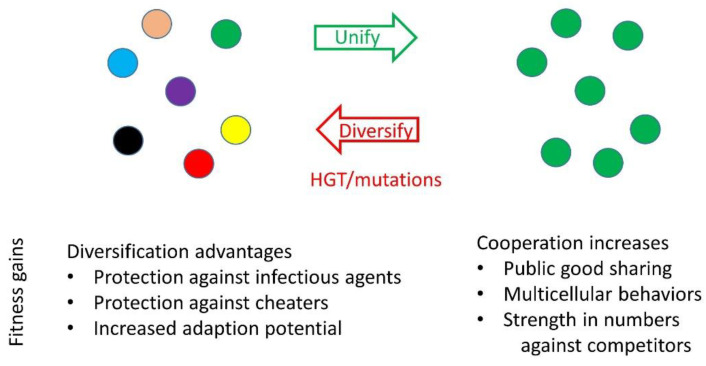
Evolution of social diversification or unification. Selective forces drive populations to become more unified or diverse. Unification forces include a dominant genotype that outcompetes related genotypes or selective pressures favoring large cooperative groups. Rapid diversification is frequently driven by HGT (Figure 2).

Successful MGEs provide a fitness benefit to the host, which in turn allows the element to propagate. For MDEs, both the element and the host benefit, but they do so at the expense of the host’s siblings (Figure 2). This leads to an underlying question: Does such social diversification benefit populations at large, or do they simply tolerate the stress? On the one hand, KD results in elevated infighting among kin and hence reduces a population’s size and fitness, making it vulnerable to larger and more cooperative competitor populations (Figure 4). Furthermore, considering competitive exclusion theory, one predicts that following diversification, the new lineages should not coexist indefinitely since they occupy the same niche [91]. In some environments, this may occur [79], while in other environments this may not happen because of the dynamics and diversity of MDE transfer [5]. 

On the other hand, diversification offers benefits (Figure 4). Empirically, when examined, new KD loci are often found in populations [5,6], thus highlighting the success of the elements and apparent fitness gains by hosts. For populations at large, diversification by KD functions as a barrier, or social moat, against cheaters and closely related competitors. Such division can also restrict the spread of deleterious infectious agents, e.g., phage and conjugating elements, by restricting spatial overlaps between groups. That is, although one group is compromised by an infection, it is less likely all groups are compromised, and thus the overall population is more resilient. Moreover, in the hypothetical absence of new KD loci, individuals in populations would continue to cooperate, even as they diversify by random mutations. Eventually, Hamilton’s rule (*rb* > *c*) would exceed a threshold, and genetic relatedness of recipients would no longer outweigh the cost to the actor. Therefore, MDEs provide social fluidity and selective forces that limit the level and size of cooperating groups. For a given situation, the optimal sizes of cooperating groups, as well their degree of relatedness, depends on the nature of their interactions, the properties of a taxa and local selective forces. Importantly, a specific benefit of fluid KD systems is that they target related individuals, which limits the size of cooperative groups and thus helps eliminate new competitors or cheaters that arise.

### 1.7. A Case Study: Rapid Social Diversification in a Local Natural Population by MDEs

To understand social diversification in nature, one needs natural isolates from a taxon that co-evolved in a particular niche. Such isolates are then tested for social compatibilities, and any incompatibilities can then be mapped to specific social genes. Unfortunately, there are few reports investigating social diversification in such a manner, nevertheless here we do outline one that involves a series of studies on *M. xanthus*.

Over 15 years ago, the Velicer group undertook a systematic approach to isolate *M. xanthus* strains from a small patch of forest soil [92]. From 100 adjacent soil plugs, they specifically sought *M. xanthus* isolates, where only one isolate was retained from each plug. In total, 78 attempts were successful. In a relatively simple social compatibility assay, they tested whether colony swarms would merge in harmony or whether they discriminated against one another, as indicated by demarcations. By analyzing a subset of strains, they found at least 45 distinct compatibility groups [9], though the total number is likely larger. These striking results revealed that *M. xanthus* isolates sharing the same habitat had diverged into many distinct social groups that actually kill their kin [5]. In separate work, draft genomes from the two largest clades were obtained [55]. These clades contained 22 of the 78 isolates and represented 11 distinct compatibility types. Their comparative genome analysis found numerous sequence differences between compatibility types that suggested a genetic basis for inter-strain incompatibilities, but the underlying mechanisms remained unknown [55].

From our work, we identified hundreds of unique KD *sitAI* loci, which in various theoretical combinations represented an astronomical number of ‘self-identity barcodes’ or kin groups [42,52]. With this information, we used comparative genomics to predict social group identities among the 22 strains with draft genomes. From this analysis of *sitAI* loci, we found a perfect correlation between unique *sitAI* loci and strains that belong to distinct compatibility types [5]. Of course, inherent in this analysis is that predictions are only possible between strains that have compatible TraA receptors that allow OME [44]. Therefore, since the clades represented two incompatible TraA receptors, there had to be a second KD mechanism to explain incompatibilities between isolates with divergent *traA* alleles. Based on prior reports [57,58], the T6SS was a plausible reservoir of KD effectors. By again using comparative genomics, we also found a perfect correlation between unique T6SS effectors and strain incompatibilities [5]. Next, we tested our predictions by constructing a series of mutants. Here, single mutations blocking OME or T6SS function did not relieve KD, but double mutants did for most strains. However, for two strains, a third KD system (Rhs), with unique effectors and apparent means of delivery, was identified [5,55], and when all three systems were inactivated, antagonism was relieved. Therefore, among strains within a clade (identical *traA* alleles), OME, T6SS and in some cases Rhs, governed KD. Finally, it is interesting to note that an HGT event occurred between these clades, presumably by transduction, whereby the *traAB* alleles were swapped. Therefore, these sub-clade isolates could no longer conduct OME with fellow clade members, but could from members of the other clade, thus dramatically changing their social interactions. 

Strikingly, genome sequences among members within a clade showed they were highly related (e.g., DNA identities >99.99%) [55], but contained islands of unique sequences. We analyzed these islands and found they contained MGEs belonging to four prophage families and one insertion element family [5]. Importantly, all of the unique OME, T6SS and Rhs effector loci mapped to these MGEs (25 unique elements in 11 genomes). Therefore, in recent evolutionary time, these MDEs invaded ancestors of these clades and triggered their rapid diversification into 11 distinct social groups as depicted in Figure 2B. However, in this small patch of forest soil, these clades likely contained many additional social groups, because only a small number of strains were practically isolated. Moreover, these combined results suggest this particular microbiome contained a large pool of MDEs (Figure 1), the origin of which is unknown. In summary, these series of studies revealed how MGEs drove the rapid social diversification of *M. xanthus* populations in their natural habitat. This resulted in diverse social groups that appear to be resilient to changing and challenging environmental conditions.

## 2. Conclusions

Social diversification plays an important role in evolution because it facilitates genetic isolation, which, as described here, often coincides with antagonism. In turn, conflict and genetic isolation can accelerate evolution and lineage divergence by promoting arms races between groups [4]. Next-generation sequencing and metagenomics have provided powerful new approaches and insights into the dynamic nature of HGT of MGEs. This, combined with isolation methods and microbial assays, allows for exciting new discoveries into the mechanisms of diversification and its consequences in natural habitats. With these and other strategies, future work will unlock the fluid nature of microbial social interactions, how they change, and the impacts they have on a population’s fitness.

## Figures and Tables

**Table 1:** KD systems and effectors found on MGEs.

MGE Type	Type of Genes Encoded	Host Organism	References
Plasmid	Colicin, T6SS, SitA toxin	*E. coli*, *Vibrio*, *Proteobacteria* (e.g., *Rhizobium*, *Ralstonia*), *Myxococcus*	[52,71,72,74,75]
Prophage	Effectors T6SS, OME, Rhs; MuF toxins, bacteriocins	*Myxococcus*, *E. coli*, widespread	[5,64,76]
Transposon	CDI	*E. coli*, *Dickeya*, *Burkholderia*	[77,78,79,80]
ICE	T6SS, toxins, bacteriocins	*Bacteroides*, *Parabacteroides*	[78,79]

## Data Availability

Not applicable.
